# Supplementary material for: Possible Repositioning of an Oral Anti-Osteoporotic Drug, Ipriflavone, for Treatment of Inflammatory Arthritis via Inhibitory Activity of KIAA1199, a Novel Potent Hyaluronidase
Source: Int J Mol Sci. 2022 Apr 7;23(8):4089. doi: 10.3390/ijms23084089 (PMC9030858; doi:10.3390/ijms23084089)
Supplement: Supplementary file 1 [file ijms-23-04089-s001.zip › ijms-1652128-supplementary.pdf]

Article

Possible repositioning of an oral anti-osteoporotic drug, Ipriflavone, for treatment of inflammatory arthritis via inhibitory activity of KIAA1199, a novel potent hyaluronidase.

Hiroshi Koike[1], Yoshihiro Nishida[2], Tamayuki Shinomura[3], Bisei Ohkawara[4], Kinji Ohno[4], Lisheng Zhuo[1], Koji Kimata[5], Takahiro Ushida[5], Shiro Imagama[1]

[1]Department of Orthopedic Surgery, Nagoya University Graduate School of Medicine, 65 Tsurumai, Showa, Nagoya, Aichi, 466-8550, Japan
[2]Department of Rehabilitation Medicine, Nagoya University Hospital, 65 Tsurumai, Showa, Nagoya, Aichi, 466-8550, Japan
[3]Department of Hard Tissue Engineering, Tokyo Medical and Dental University, Tokyo, 113-8510, Japan
[4]Division of Neurogenetics, Center for Neurological Diseases and Cancer, Nagoya University Graduate School of Medicine, 65 Tsurumai, Showa, Nagoya, Aichi, 466-8550, Japan
[5]Multidisciplinary Pain Center, Aichi Medical University, Nagakute, Aichi, 480-1195, Japan

* Correspondence: Correspondence and reprint requests to Y. Nishida, Department of Rehabilitation Medicine, Nagoya University Hospital, 65 Tsurumai, Showa, Nagoya, Aichi, 466-8550, Japan
Phone: 81-52-741-2111, Fax: 81-52-744-2260, E-mail: ynishida@med.nagoya-u.ac.jp

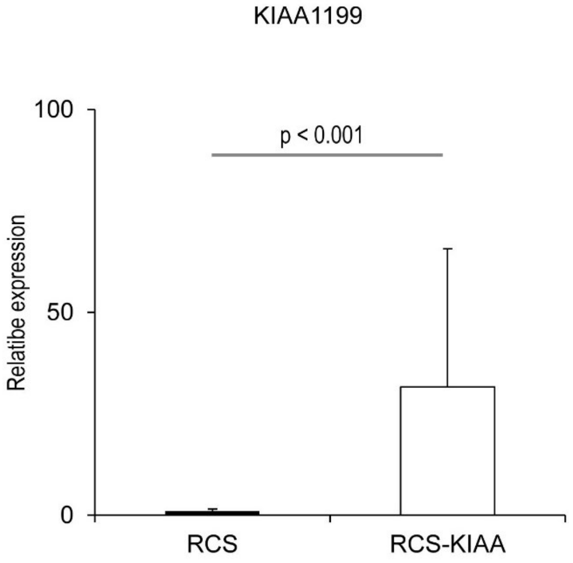

Figure S1. Relative mRNA expression of KIAA1199 in RCS cells. RCS-KIAA represents RCS cells, which are transfected with mouse KIAA1199 cDNA.

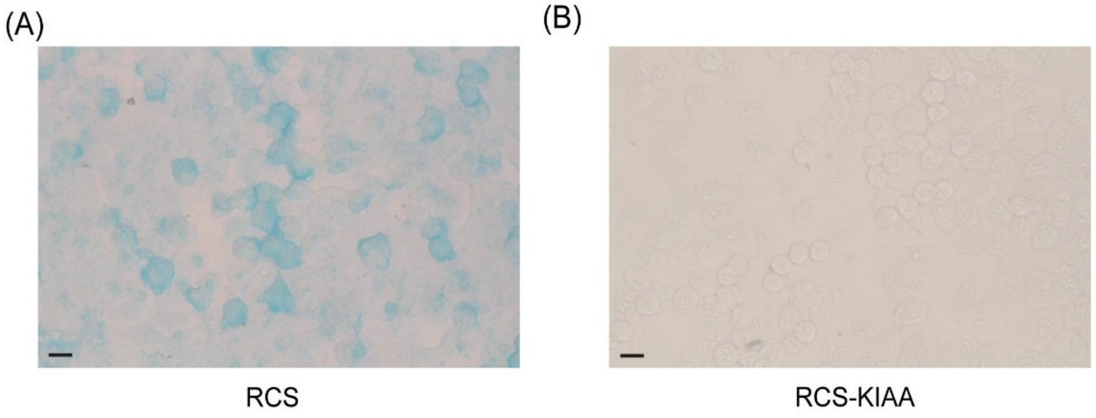

Figure S2. Representative image of RCS with Alcian blue staining RCS cells (A) and RCS-KIAA cells (B) were mono-cultured for 24 h and stained with Alcian blue. Bar = 20 μm.

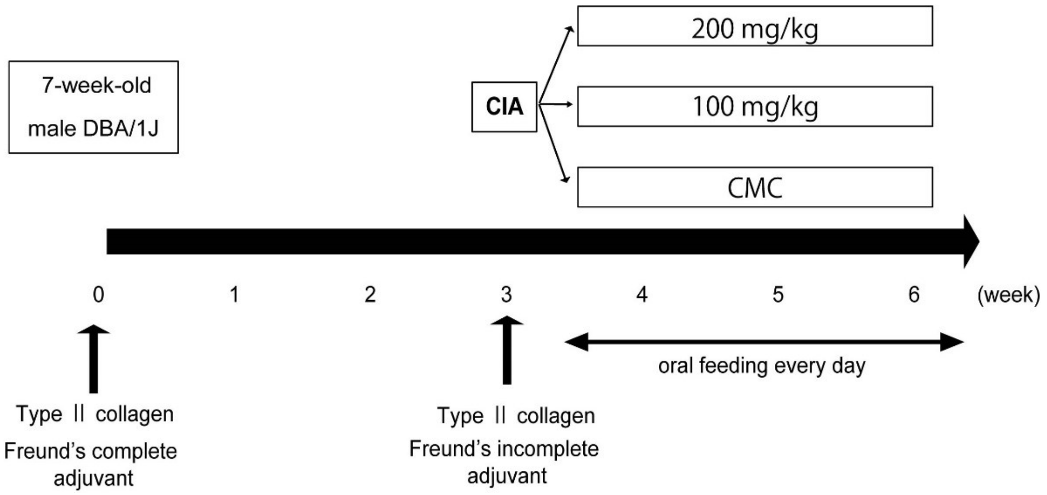

Figure S3. Schematic diagram of the experimental protocol for the CIA mouse model.
